# Epigenetic mechanisms and therapeutic advances in diffuse midline glioma (Review)

**DOI:** 10.3892/or.2026.9055

**Published:** 2026-01-20

**Authors:** Wenbo Wu, Wenlin Chen, Wenbin Ma, Yu Wang

**Affiliations:** Department of Neurosurgery, Peking Union Medical College Hospital, Chinese Academy of Medical Sciences and Peking Union Medical College Hospital (East), Beijing 100730, P.R. China

**Keywords:** diffuse midline glioma, epigenetic modifications, histone, targeted therapy, immunotherapy

## Abstract

Gliomas are the most common primary malignant tumors of the central nervous system in adults, with diffuse midline gliomas (DMG) being particularly aggressive and associated with inferior survival rate. Notwithstanding advances in molecular diagnostics and epigenetics, the specific pathological mechanisms of DMG remain to be fully elucidated. A series of studies have demonstrated that histone modifications, particularly the histone H3 lysine 27 (H3K27)M mutation, play a pivotal role in the development and progression of DMG. The mutation disrupts histone methylation and acetylation to induce widespread gene expression abnormalities, tumor aggressiveness and treatment resistance. Conventional treatments such as surgery, local radiotherapy and chemotherapy offer limited efficacy. However, emerging precision therapies targeting histone mutations, epigenetic modifications and innovative immunotherapies show promise in improving outcomes. The present study provided a comprehensive overview of the molecular mechanisms, epigenetic characteristics and the latest therapeutic advances in DMG. By investigating the H3K27M mutation and its associated epigenetic mechanisms, the present review aimed to establish theoretical frameworks and research avenues for developing precise therapeutic strategies for DMG, thus contributing to advancing the field of personalized medicine.

## 1. Introduction

Gliomas are the most prevalent primary malignant tumors of the central nervous system (CNS) in adults (1), with diffuse midline glioma (DMG) standing out for its high invasiveness and abysmal prognosis. Driven by the histone H3 lysine 27 (H3K27)M mutation, DMG exhibits aggressive behavior and marked resistance to treatment, making it a formidable challenge in neuro-oncology.

DMG predominantly affects children and adolescents, whereas adult cases are uncommon. Clinically, numerous pediatric DMGs present as diffuse intrinsic pontine glioma (DIPG), a radiographic entity centered in the pons. Epidemiologic estimates suggest 20–30 new DIPG cases annually in the UK (2) and 100–400 annually in the US (2,3) and a Canadian population-based cohort identified 143 pediatric DIPG cases over a 10-year period (~14 cases per year) (2). DMG remains highly lethal, with a median overall survival (OS) of ~11 months (4) and 1-, 2- and 5-year survival rates of 42.3, 9.6 and 2.2% in a 1,008-case registry cohort (5).

DMGs are invasive tumors affecting midline structures such as the thalamus, pons, brainstem and spinal cord. Clinical manifestations vary by location, including cranial nerve dysfunction, ataxia and raised intracranial pressure. MRI is key for diagnosis, showing heterogeneous features such as diffuse or distinct boundaries, variable contrast enhancement and similarities to low-grade astrocytomas (6). Pons-involved DMGs often infiltrate >50% of the pons and may encase the basilar artery, with minimal T1 contrast enhancement and uneven T1/T2 signal intensities, often with necrosis or hemorrhage (7,8), reflecting their histopathological heterogeneity.

Molecular pathological studies of DMG have revealed its unique epigenetic characteristics, focusing on the pivotal role of the H3K27M mutation in its pathogenesis (9). This mutation replaces lysine 27 of histone H3 with methionine, substantially reducing H3K27 trimethylation (H3K27me3) and causing widespread gene expression reprogramming. It disrupts epigenetic regulation, promoting tumor proliferation, invasion and treatment resistance (10). Additionally, the H3K27M mutation dysregulates multiple key signaling pathways, including MAPK and PI3K/AKT, driving rapid growth and creating a tumor-supportive micro-environment (11). These findings establish H3K27M mutation as a central driver of DMG and underscore the urgent need for targeted therapeutic strategies.

Despite notable advances in understanding the molecular basis of DMG, the therapeutic landscape for this disease remains beset with formidable challenges. Surgery, radiotherapy and chemotherapy provide minimal improvement in overall survival currently, as epigenetic alterations driven by the H3K27M mutation render these tumors highly resistant to conventional treatments. However, emerging targeted therapies and immunotherapies, including CAR-T cell therapy, have garnered considerable research interest (12) though they require further clinical validation.

Against this backdrop, the present review systematically summarized the molecular characteristics, genetic signatures and latest progress in DMG treatment, emphasizing the effect of histone alterations and innovative therapeutic strategies to deepen the understanding of DMG and provide theoretical support and guidance for future research.

## 2. Modifications to the definition of DMG

The definition of DMG has evolved markedly with the 2016 and 2021 World Health Organization (WHO) CNS tumor classifications, shifting towards molecular pathology-based diagnosis.

### Classification and definition in WHO before 2016

DMG, termed diffuse intrinsic pontine glioma (DIPG) before 2016, was first defined by Wilfred Harris in 1926 for its pontine location and histological features (13,14), predominantly affecting children aged 5–10 with a dismal prognosis (median OS less than a year and five-year survival rate <1%) (15). Traditional classification could not distinguish subtypes or elucidate molecular mechanisms (16).

The 2016 WHO classification introduced molecular diagnostics, defining DMG by the H3K27M histone mutation, specifically the K27M mutation in the H3F3A, HIST1H3B and HIST1H3C genes (17,18). This mutation disrupts lysine 27 methylation of histone H3, altering gene regulation and driving tumorigenesis (19), thereby enhancing diagnostic precision and differentiation.

### Updates in the 2021 WHO classification

The 2021 WHO classification renamed ‘Diffuse Midline Glioma, H3K27M-mutant’ to ‘Diffuse Midline Glioma, H3 K27-altered’ to encompass broader molecular changes (20), including EZHIP overexpression (21,22), which reduces H3K27me3 levels, promoting invasiveness and therapy resistance (23). These updates improved diagnostic accuracy, highlighted tumor heterogeneity and facilitated tailored, patient-specific treatment strategies to enhance outcomes (24).

## 3. Significance of histones in cancer

### Definition and functions of histone and oncohistones

Histones are the primary structural proteins of chromatin, packaging DNA into nucleosomes comprising two copies each of H2A, H2B, H3 and H4. Beyond DNA condensation, histones regulate gene expression through post-translational modifications such as methylation, acetylation, phosphorylation and ubiquitination.

Occurring on lysine residues such as H3K4, H3K27 and H4K20, methylation can activate or repress transcription based on its site and extent (mono-, di-, or trimethylation) (25). This process is dynamically regulated by histone methyltransferases (such as SETD1A, which activates transcription via H3K4 methylation) and histone demethylases (such as KDM1A, which represses transcription by demethylating H3K4me2/1) (26). Catalyzed by histone acetyltransferases (HATs) such as p300/CBP, acetylation neutralizes the positive charge of lysine, reducing the electrostatic interaction of histones with the negatively charged DNA (27), loosening chromatin and facilitating transcription. Phosphorylation is closely associated with cell cycle regulation, DNA damage response and transcriptional activation (28,29). Modifications, such as H2AX serine 139 phosphorylation, signal DNA damage, recruit repair proteins and coordinate cell cycle responses (30). Ubiquitination, primarily on H2A and H2B, affects chromatin structure and gene regulation, depending on the site and mono- or poly-ubiquitination status (31).

Oncohistones, a subset of histones predominantly observed in H3 (32,33), drive tumorigenesis and cancer progression by disrupting chromatin structure (34). Mutations alter key modifications such as H3K27 acetylation, which activates oncogenes such as MYC and epidermal growth factor receptor (EGFR) (35) and H3K27me, which silences tumor suppressors by compacting chromatin and blocking transcription factor access. Elevated H3K27me levels are linked to silenced tumor suppressors in breast, liver and lung cancers (36–38). These changes promote tumor proliferation, invasiveness and resistance, making oncohistones pivotal therapeutic targets.

### Systemic effects of histone modifications

Histone modifications orchestrate key pathways such as PI3K/AKT/mammalian target of rapamycin (mTOR) and MAPK/ERK (39,40), influencing tumor proliferation, apoptosis and drug resistance (41). Aberrant modifications disrupt gene networks, suppress immune responses and drive metastasis, as seen with elevated HIST1H2BJ activating MMP9 and VEGF in breast cancer brain metastases (42,43). These changes highlight the critical role of histone modifications in cancer progression and metastasis.

## 4. H3K27M mutation in the development of DMG

**Figure 1.**
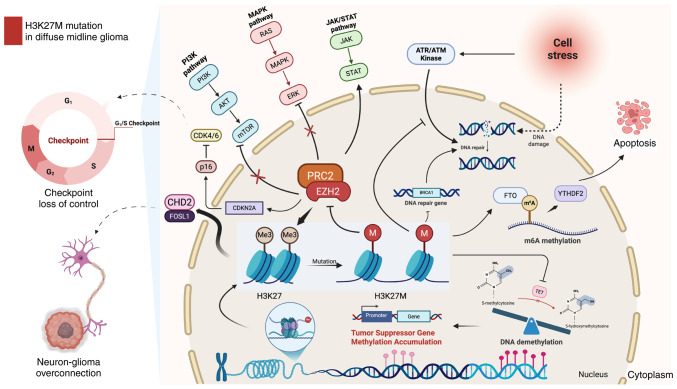
H3K27M mutation in diffuse midline glioma. The H3K27M mutation markedly reduces H3K27me3 levels by inhibiting the EZH2 methyltransferase activity of the PRC2 complex, leading to disrupted methylation regulation of tumor suppressor genes and inducing widespread transcriptional abnormalities. This mutation promotes uncontrolled cell cycle progression, exemplified by dysregulation of the CDK4/6-p16 pathway and enhances tumor cell proliferation and invasion through the activation of key signaling pathways, including MAPK/ERK, PI3K/AKT/mTOR and JAK/STAT. The H3K27M mutation also induces DNA repair deficiencies by impairing the homologous recombination repair and base excision repair pathways. It also affects the cellular stress response by altering the ATR/ATM kinase pathway, compromising the cell's ability to manage DNA damage. These defects exacerbate genomic instability and transcriptional dysregulation, further contributing to tumor progression. The H3K27M mutation alters m6A methylation and FTO activity, making DMG cells sensitive to FTO inhibition, which disrupts cell cycle gene expression and induces apoptosis. Furthermore, the CHD2/FOSL1 axis plays a crucial role in abnormal synaptic interactions between neurons and tumor cells, enhancing tumor growth and invasion by promoting excessive neuronal-tumor connectivity. Figure created using BioRender.com. H3K27, histone H3 lysine 27, H3K27me3, H3K27 trimethylation; PRC2, polycomb repressive complex 2; mTOR, mammalian target of rapamycin; FTO, fat-mass- and obesity-associated protein; DMG, diffuse midline gliomas.

Diffuse midline glioma is defined by the H3K27M oncohistone, which disrupts methylation at histone H3 lysine 27 (H3K27). This missense substitution (lysine-to-methionine) occurs in H3.1 or H3.3 variants and is associated with a profound reduction of H3K27me3, a key epigenetic mark of transcriptional repression. The resulting epigenetic reprogramming reshapes chromatin accessibility and transcriptional programs, thereby promoting aggressive phenotypes, including uncontrolled proliferation, invasion and treatment resistance (44) (Fig. 1).

### Inhibition of H3K27me3 by H3K27M

H3K27 trimethylation is primarily catalyzed by the EZH2 SET domain within the Polycomb Repressive Complex 2 (PRC2), which maintains Polycomb-mediated transcriptional repression programs involved in lineage control and proliferation. Structural and biochemical studies (45) show that the H3K27M oncohistone engages the PRC2 catalytic groove: the substituted methionine inserts into the lysine-access channel of the EZH2 SET domain, thereby competitively inhibiting PRC2 methyltransferase activity and exhibiting markedly higher affinity than the corresponding wild-type H3K27 peptide [equilibrium dissociation constant (K_d_) ~3.3±0.4 µM for H3K27M compared with ~52±12 µM for H3K27]. This inhibitory mode provides a mechanistic basis for the global reduction and redistribution of H3K27me3/PRC2 and the ensuing transcriptional reprogramming observed in pediatric H3K27-altered high-grade gliomas (44).

The suppression of H3K27me3 by H3K27M alters gene expression and activates multiple signaling pathways essential for cell proliferation, survival and invasion. In the PI3K/AKT/mTOR axis, growth-factor/RTK inputs activate PI3K and generate PIP3, recruiting and activating AKT, which phosphorylates downstream effectors to promote cell-cycle progression and survival while suppressing apoptosis (39); mTOR further sustains biosynthetic and metabolic programs that facilitate rapid tumor expansion (46).

In parallel, aberrant activation of the JAK/STAT pathway can reinforce proliferative transcriptional programs and contribute to immune-evasive phenotypes within the tumor microenvironment, as JAK-dependent STAT phosphorylation promotes nuclear transcription of genes linked to growth and immunoregulation (44,47).

Moreover, MAPK/ERK signaling, often downstream of RTKs, can be potentiated, enabling RAS-RAF-MEK-ERK propagation and ERK-driven nuclear transcriptional outputs that promote proliferation and therapy resistance (48).

### Extensive changes in DNA methylation

The H3K27M mutation disrupts histone methylation and induces widespread changes in DNA methylation. Research by Vanan *et al* (49) and Graham *et al* (50) revealed that reduced H3K27me3 in H3K27M mutant cells extends across the genome, altering DNA methylation beyond the sites of mutant histone localization. This indicates that the mutation indirectly affects the regulation of numerous genes.

Further studies by Paine *et al* (51) demonstrated significant DNA methylation changes, particularly in the promoter regions of tumor suppressor genes such as TP53 and RB1, leading to their silencing and facilitating the malignant progression of tumors.

Typically, the TET family of enzymes (TET1, TET2 and TET3) oxidizes 5-methylcytosine (5mC) to 5-hydroxymethylcytosine (5-hmC) in a process known as DNA demethylation (52). However, the H3K27M mutation inhibits TET enzyme activity by disrupting chromatin-associated factors, reducing TET recruitment and markedly decreasing 5-hmC levels. This results in the accumulation of DNA methylation, particularly in the promoter regions of tumor suppressor genes (53,54), creating an epigenetic landscape that promotes DMG invasiveness and proliferation.

### Regulation of m6A RNA methylation

Recent research (55) reveals that H3K27M-mutant DMG exhibits unique m6A RNA methylation patterns, with m6A marks enriched on key cell cycle genes. The m6A demethylase fat-mass- and obesity-associated protein (FTO) is critical for DMG cell proliferation; inhibition of FTO (such as by FB23-2) increases m6A levels, disrupts cell cycle progression, reduces tumor cell survival and induces apoptosis. These effects are partly mediated by the m6A reader YTHDF2, which is highly expressed in DMG and promotes degradation of cell cycle transcripts. Targeting FTO-driven m6A dynamics thus represents a promising therapeutic strategy for DMG.

### Cell cycle regulation by H3K27M mutation

The H3K27M mutation disrupts cell cycle regulation by inhibiting the activity of the PRC2 complex and silencing tumor suppressor genes such as RB1 and CDKN2A. This leads to the loss of G_1_/S checkpoint control, allowing cells to bypass critical regulatory mechanisms and proliferate uncontrollably (56) (Fig. 1).

Williams *et al* (57) highlighted the pivotal role of the p16 protein, encoded by CDKN2A, in maintaining cell cycle control by inhibiting CDK4/6 activity and preventing the transition from the G_1_ to S phase. In H3K27M mutant tumor cells, the loss of p16 function results in cell cycle imbalance and accelerated tumor growth.

### H3K27M mutation inhibits DNA repair

The H3K27M mutation additionally impairs DNA repair processes, exacerbating genomic instability. In normal cells, DNA damage is repaired to preserve genomic integrity. However, in H3K27M mutant cells, the capacity for DNA repair, particularly in the homologous recombination repair and base excision repair pathways, is markedly compromised (Fig. 1). The expression of essential DNA repair genes, including BRCA1, is notably diminished, rendering them prone to accumulating genetic mutations and facilitating tumor progression (58). Furthermore, H3K27M rewires the DNA damage response (DDR) and checkpoint circuitry (including ATM/ATR-linked pathways), which can contribute to treatment resistance but also creates actionable vulnerabilities for DDR-targeted radio sensitization strategies (59).

### Neuron-tumor cell interactions

Beyond epigenetic changes, the H3K27M mutation promotes tumor expansion by regulating neuron-tumor interactions (Fig. 1). Chromodomain-Helicase-DNA-binding protein 2 (CHD2) plays a crucial role in chromatin remodeling within the neuronal-tumor microenvironment, regulating genes essential for synaptic formation, neuronal excitability and nerve transmission, such as SYP and PSD95, which maintain synaptic integrity (60).

In H3K27M mutant cells, CHD2 remodels chromatin accessibility and drives an axon-guidance/synaptic transcriptional program; mechanistically, a FOSL1-CHD2 regulatory axis promotes neuron-glioma interactions and neuron-induced tumor proliferation (60). Upregulation of axon-guidance and synaptic gene programs strengthens neuron-glioma interactions and neuron-induced proliferation in H3K27-altered DMG.

## 5. Other histone modifications in DMG

Beyond the dysregulation of H3K27 trimethylation by the H3K27M mutation, other histone modifications markedly contribute to DMG progression. These modifications regulate chromatin structure and gene expression, driving tumor cell proliferation, differentiation and invasion (61). Critical histone modifications and their roles in DMG are outlined below.

### H3K27ac

H3K27ac, the acetylation of lysine 27 on histone H3, is associated with active chromatin states, promoting gene transcription by maintaining chromatin openness, predominantly at promoters and enhancers.

In DMG, the H3K27M mutation indirectly increases H3K27ac by inhibiting H3K27me3, activating oncogenes such as MYC and PDGFRA, which drive tumor growth. Additionally, this upregulation keeps chromatin at promoter and enhancer regions open, facilitating transcription factors such as SP1 and AP-1 binding (62). Menez *et al* (56) demonstrated that HATs such as p300 and CBP reinforce this open state, creating a positive feedback loop that amplifies the expression of genes promoting proliferation, survival and invasion.

Mota *et al* (63) found that the SWI/SNF chromatin remodeling complex, particularly the BAF subtype, contributed to H3K27ac accumulation. The antagonistic interaction between BAF and PRC2 enhances the effects of H3K27ac. Targeting this pathway with AU-15330, which is a PROTAC compound that degrades SMARCA4, SMARCA2 and PBRM1, effectively reduces H3K27ac levels, impairing chromatin openness and inhibiting tumor proliferation.

### H3K9me3

H3K9me3 is a repressive histone mark enriched in heterochromatin regions, suppressing gene transcription through chromatin compaction. In DMG, altered H3K9me3 levels are associated with silencing tumor suppressor genes.

Xin *et al* (64) revealed that the histone methyltransferase SUV39H1 generates H3K9me3 marks, recruiting protein complexes to compact chromatin and repress tumor suppressor genes such as CDKN1A, CDKN2B and CDKN2D, thereby promoting tumor cell proliferation. The fungal metabolite Chaetocin, a SUV39H1 inhibitor, reduces H3K9me3 levels, which restores tumor suppressor gene expression and induces apoptosis in DMG cells.

Notably, when H3K9me3 levels are inhibited, DMG cells activate compensatory pathways such as the dopamine receptor D2 (DRD2) pathway to sustain tumor growth. A combination therapy of Chaetocin and DRD2 antagonists (such as ONC201) has been shown to enhance anti-tumor effects and reduce treatment resistance (64).

### H3K36me3

H3K36me3 is an activating histone mark involved in transcriptional elongation and DNA repair. The H3.3G34R/V mutation reduces H3K36me3 levels (51), disrupting transcriptional elongation and mismatch repair, contributing to a mild ‘hyper-mutator’ phenotype by impairing DNA repair mechanisms (65).

The loss of H3K36me3 compromises genomic stability, allowing the accumulation of mutations that drive tumorigenesis. These findings highlight the essential role of H3K36me3 in maintaining cellular function and its dysregulation as a key factor in cancer progression.

Given the pivotal role of these epigenetic modifications in DMG pathogenesis, targeting these epigenetic alterations presents a promising avenue for developing novel therapeutic strategies to combat DMG.

## 6. Current treatment regimens

Local treatments, primarily encompassing surgery and radiotherapy, are central to managing DMG. However, the invasive nature of DMG and its midline location limit their effectiveness, underscoring the need for more effective therapeutic options.

### Surgical treatments

Surgery for DMG aims to obtain tissue for diagnosis and molecular analysis, as its midline location makes complete resection highly risky. Biopsies, particularly stereotactic ones, highlighted in CPN Paris 2011, are vital for identifying mutations such as H3K27M, enabling targeted therapies (66). Advances in minimally invasive techniques and molecular profiling have made biopsy a key clinical tool, offering critical insights with minimized risks (67).

A multicenter study revealed no survival benefit from resection over biopsy in H3K27M-mutant DMG patients, with OS at 12 months for resection and 11 months for biopsy (68). However, resection was associated with longer ICU stays and higher neurological deficits. Moreover, the surgical intervention did not improve the Karnofsky Performance Status (68,69). Similarly, a systematic review reported a 13.02% complication rate for stereotactic biopsy, with no biopsy-related deaths and a diagnostic success rate of 97.4% (69). While biopsies do not markedly extend survival, they provide invaluable molecular data for personalized therapies.

### Radiotherapies

#### External beam radiotherapy (EBRT)

EBRT remains the standard treatment for DMG, delivering 54–59.4 Gy over 30–33 sessions (70). While it temporarily alleviates symptoms and delays progression for 6–12 months, recurrence is almost inevitable within a year, with a median OS of 8–12 months (71).

### Hyper-fractionated radiotherapy and hypo-fractionated radiotherapy

Alternative approaches have been tested, such as hyper-fractionated (e.g., 70.2 Gy in smaller fractions) and hypo-fractionated (e.g., 39 Gy in larger fractions) radiotherapy, but have shown no significant survival benefits. Median survival remains 7.8–8.5 months (71,72), with logistical benefits being the primary advantage.

### Proton radiotherapy

Proton therapy minimizes damage to the healthy brain, particularly in pediatric patients, but does not markedly improve long-term survival. Re-radiation after recurrence offers limited benefits and poses a higher risk of radiation-induced damage (73), such as neurological and cognitive impairments (74,75).

### Limitations of radiotherapy

Despite temporary relief, tumor progression is inevitable, with progression-free survival (PFS) of only 6–8 months and a 5-year survival rate <1% (76,77). Combining radiotherapy with agents such as Bromodomain and Extra-Terminal motif (BET) inhibitors or epigenetic drugs (such as chitosan) shows potential in augmenting its tumor-killing effects by disrupting DNA repair (66,78).

### Chemotherapies

Chemotherapy serves as a complementary treatment for DMG, largely as an adjunct to radiotherapy when resection is not feasible. However, across prospective and registry-era experiences, conventional cytotoxic chemotherapy has not produced a consistent survival benefit beyond radiotherapy alone and its use is increasingly framed within clinical trials or rational combinations rather than as stand-alone escalation (Table I).

### Temozolomide

TMZ is an oral alkylating agent whose key cytotoxic lesion is O6-methylguanine, which can trigger replication-associated mismatch repair-dependent DNA damage and apoptosis. In a UK phase II study evaluating dose-dense TMZ with standard radiotherapy for classical DIPG/DMG, median OS was 9.5 months, with 1-year OS of 35% and the regimen did not demonstrate a survival benefit over radiotherapy alone; hematologic toxicity led to treatment withdrawal and frequent dose reductions (79) (Table I). Similarly, radiotherapy with concurrent and adjuvant TMZ yielded median OS of 11.7 months and 1-year OS of 50%, but no significant improvement in outcome and higher hematologic toxicity compared with radiotherapy alone (80). A major biological constraint is O6-methylguanine-DNA methyltransferase (MGMT)-mediated repair of TMZ-induced O6 lesions: H3K27M-altered DMG frequently shows lack of MGMT promoter methylation and increased MGMT expression, which is associated with TMZ resistance in DMG models (81). Accordingly, pharmacologic MGMT inhibition, such as O6-benzylguanine, has been limited by increased hematologic toxicity and often necessitates substantial TMZ dose reduction, constraining clinical utility (82,83). Studies (84–86) demonstrate that BET inhibitors, as well as other epigenetic modulators, including histone deacetylase (HDAC) and EZH2 inhibitors, can sensitize DMG cells to TMZ, with preclinical evidence supporting synergistic antitumor effects when combined.

### Cisplatin and etoposide

Cisplatin primarily induces cytotoxicity by forming DNA cross-links, whereas etoposide inhibits topoisomerase II and triggers DNA double-strand breaks; together they provide a rationale for combination regimens that converge on DNA-damage-driven apoptosis.

In a mono-institutional 20-year experience of pediatric diffuse pontine gliomas treated with multi-agent therapy incorporating cisplatin/etoposide (plus isotretinoin and others), the reported median survival was 7 months and the 1-year survival rate was 45% (87). Clinically, limited benefit is assumed to be related in part to inadequate CNS drug exposure under an intact/tight blood-brain barrier (BBB) in DMG (88), although rare long survivors exist. For example, a pineal-region DMG case with >6-year survival after multimodal management in which initial carboplatin-etoposide was ineffective but extensive resection, high-dose radiotherapy and bevacizumab-based maintenance was associated with durable control (89).

### Gemcitabine

Gemcitabine is a cytidine analog that inhibits DNA synthesis via DNA polymerase and ribonucleotide reductase and has been explored as a radiosensitizer in DMG (9,90).

In a phase I/II trial of weekly gemcitabine with radiotherapy, treatment was safe and well tolerated, with PFS 4.8 months and median OS 8.7 months (90); Quality of life tended to improve, but survival was not superior to historical controls, supporting mainly palliative value.

### Capecitabine

Capecitabine is an oral prodrug of 5-fluorouracil; because radiation can induce thymidine phosphorylase, capecitabine has been tested as a potential RT-sensitizing strategy in DMG (91). In the PBTC phase Il study (91), capecitabine + RT was tolerable, but outcomes were not improved: 1-year PFS 7.21 vs. 15.59% in the historical control (P=0.007) and no OS difference (P=0.30), leading to the conclusion that capecitabine did not improve prognosis in newly diagnosed DMG.

### Irinotecan

Irinotecan is a topoisomerase I inhibitor that stabilizes the cleavage complex and induces lethal DNA strand breaks, thereby suppressing tumor proliferation (92). In a phase I trial, Krystal *et al* (93) evaluated irinotecan plus bevacizumab and mebendazole in 10 pediatric/AYA HGG patients, including 7 DMG, reporting no dose limiting toxicities and a PFS and OS of 4.7 and 11.4 months from treatment initiation; the overall response rate was 33% (two PRs and one CR sustained for 10 months).

### Cyclophosphamide

Cyclophosphamide is an alkylating pro-drug that generates phosphoramide mustard, producing DNA cross-links and triggering apoptosis (94).

In a phase I trial of intravenous GD2. CAR T cells, cyclophosphamide (with fludarabine) was used for standard lymphodepletion in 11 patients and therapy was delivered without dose-limiting toxicity. The C7R-GD2.CAR T cohort developed grade 1 TIAN in 7/8 and achieved transient neurologic improvement (2 to >12 months), with partial responses in 2/7 DMG patients by iRANO (95).

Overall, conventional chemotherapies have shown limited and largely non-practice-changing benefit in DMG. Future efforts should prioritize rational combinations (such as chemo with targeted/epigenetic or immunotherapies) and trial designs that emphasize clinically meaningful endpoints, including neurologic function and quality of life.

### Targeted therapies

**Figure 2.**
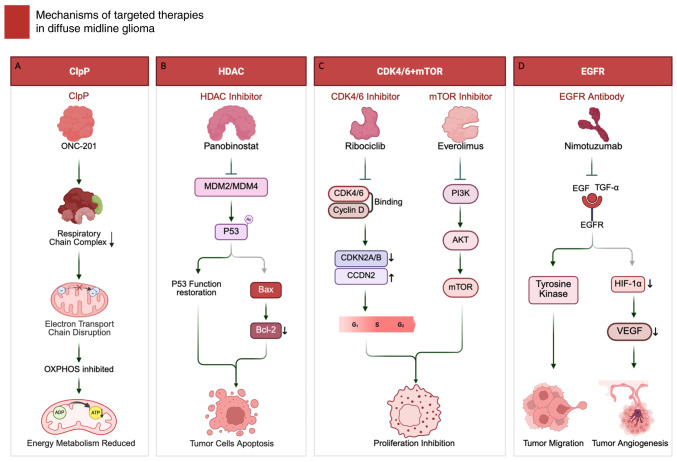
Mechanisms of targeted therapy in diffuse midline glioma. (A) ClpP agonist ONC-201 hyperactivates mitochondrial protease ClpP, leading to respiratory chain protein degradation, electron transport chain disruption, OXPHOS inhibition and reduced ATP production. (B) HDAC inhibitor panobinostat restores p53 function through MDM2/MDM4 modulation and enhanced p53 acetylation, upregulating pro-apoptotic Bax while downregulating anti-apoptotic Bcl-2 to induce tumor cell apoptosis. (C) CDK4/6 inhibitor ribociclib blocks CDK4/6-cyclin D interaction, inducing G_1_ arrest; mTOR inhibitor everolimus suppresses PI3K/AKT/mTOR signaling to inhibit proliferation and metabolism. (D) Anti-EGFR antibody nimotuzumab blocks EGF/TGF-α binding, inhibiting downstream tyrosine kinase signaling and HIF-1α/VEGF-mediated angiogenesis. Figure created using BioRender.com. ClpP, caseinolytic protease P; HDAC, histone deacetylase; mTOR, mammalian target of rapamycin; EGFR, epidermal growth factor receptor; HIF-1α, hypoxia-inducible factor 1-alpha.

Targeted therapy for diffuse midline glioma aims to exploit actionable biological dependencies that sustain tumor growth. Current strategies include mitochondrial stress-based approaches centered on caseinolytic protease P (ClpP)/imipridones such as ONC201, epigenetic modulation such as HDAC inhibitors, panobinostat for epigenetic regulation and combination therapies targeting CDK4/6 and mTOR and receptor-directed interventions focusing on EGFR inhibition. These modalities are being evaluated to counteract core metabolic/epigenetic vulnerabilities and aberrant signaling programs in DMG (Fig. 2; Table I).

### ClpP

ClpP, a mitochondrial serine protease, forms the ClpXP complex to maintain mitochondrial proteostasis. Pharmacologic hyperactivation of ClpP can drive excessive proteolysis of respiratory-chain proteins, leading to electron transport chain disruption, OXPHOS inhibition, ATP depletion and apoptotic cell death (96).

ONC201, an imipridone, acts as an allosteric ClpP agonist and antagonizes DRD2. In H3K27M-altered DMG models, ONC201-ClpP engagement promotes degradation of key respiratory chain components, collapses mitochondrial bioenergetics (ETC/OXPHOS) and suppresses tumor energy metabolism-features that match the present study's mechanistic schematic (96,97) (Fig. 2A). Downstream, ONC201 induces an ATF4/CHOP-linked integrated stress response and can promote DR5/TRAIL-mediated apoptosis (98), with reported modulation of Akt/ERK signaling consistent with a pro-apoptotic shift (99).

Clinically, integrated analysis of ONC201-treated H3K27M-mutant DMG reported longer survival when initiated after radiotherapy but before recurrence (median OS 21.7 months; PFS 7.3 months) compared with initiation after recurrence (median OS 9.3 months; PFS 3.4 months), with molecular correlates including increased 2-hydroxyglutarate and partial restoration of H3K27me3 (100). A randomized, double-blind phase 3 trial (101) is ongoing to evaluate ONC201 as post-radiotherapy maintenance therapy in newly diagnosed H3 K27M-mutant diffuse glioma, with OS and PFS as primary endpoints.

### HDAC

HDAC inhibitors reprogram chromatin by increasing histone acetylation and can reactivate the MDM2/MDM4-p53 axis, in part by reducing MDM2/MDM4 repression and enhancing p53 acetylation/transcriptional activity, thereby promoting apoptosis (102). Downstream, they upregulate pro-apoptotic genes such as Bax while downregulate anti-apoptotic genes such as Bcl-2, activating the intrinsic apoptotic pathway (103) (Fig. 2B).

Panobinostat is a multi-HDAC inhibitor with strong preclinical rationale in DMG, but systemic delivery is constrained by exposure/toxicity and uncertain BBB penetration, motivating local delivery approaches (104).

In a phase I/II trial by Su *et al* (105), panobinostat combined with radiotherapy and voriostat, followed by maintenance, was well-tolerated but showed limited survival benefits, with a 1-year PFS of 5.85% and OS of 39.2%. By contrast, PNOC015 using repeat CED of MTX110 reported median OS of 26.1 months and median PFS 7 months, supporting intra-tumoral HDAC inhibition with reduced systemic exposure (104). In adults, compassionate-use panobinostat showed good tolerability with median OS 42 months and median PFS 19 months in a small series, suggesting potential context-dependence that requires prospective validation (106).

### Cyclin-dependent kinases 4 and 6 (CDK4/6) + mTOR

CDK4/6 regulate the G_1_-to-S phase transition by phosphorylating retinoblastoma (RB) protein via cyclin D, activating E2F transcription factors and driving cell cycle progression (107,108). The mTOR in the PI3K/Akt pathway governs metabolism, protein synthesis and proliferation, with aberrations frequently observed in DMG, contributing to unchecked tumor proliferation and metabolic dependency (107).

Ribociclib, a CDK4/6 inhibitor, halts cell cycle progression at the G_1_ phase by disrupting CDK4/6-cyclin D interaction (109) (Fig. 2C), targeting CDKN2A/B deletions and CCND2 amplifications seen in DMG. Meanwhile, everolimus, which is a mTORC1 inhibitor, suppresses tumor proliferation, glycolysis and angiogenesis (110), particularly in tumors with H3.1 mutations linked to PIK3CA or PIK3R1 alterations (40). The combination of ribociclib and everolimus provides dual inhibition of cell cycle and metabolic pathways, enhancing antitumor effects with reduced overlapping toxicity.

A phase I/II study of post-radiotherapy single-agent ribociclib reported feasibility with myelosuppression as the main severe toxicity and a median OS of 16.1 months in DIPG/DMG, reinforcing ongoing interest in rational combinations and biomarker-guided selection (111). As additional supportive evidence for CDK4/6 targeting in this setting, A phase I trial assessed this combination in 19 pediatric DMG patients following radiotherapy, establishing phase II doses of 170 mg/m^2^ for ribociclib and 1.5 mg/m^2^ for everolimus (112). The median OS was 13.9 months, with 12-, 24- and 36-month survival rates of 53.3, 38.9 and 38.9%, respectively. These promising results warrant further investigation of this approach.

### EGFR

EGFR is a receptor tyrosine kinase that propagates mitogenic and pro-survival signals mainly through the RAS-RAF-MEK-ERK and PI3K-AKT cascades, thereby promoting proliferation, invasion and treatment resistance (113). Additionally, EGFR pathway activity intersects with hypoxia/angiogenic programs, in part via PI3K/AKT-hypoxia-inducible factor 1-alpha (HIF-1α)-VEGF, providing a rationale for EGFR-directed strategies to suppress both tumor growth and angiogenesis (114).

Nimotuzumab, an anti-EGFR monoclonal antibody, blocks EGF/TGF-α-EGFR engagement and thereby attenuates downstream oncogenic signaling; clinically, however, its benefit has been inconsistent (115). It induces G_1_ phase arrest, preventing tumor cell progression into the S phase (116). Additionally, it destabilizes HIF-1α, suppresses VEGF transcription and reduces angiogenesis, collectively curbing tumor growth and metastasis (117) (Fig. 2D).

A phase III trial in 42 DMG patients evaluated nimotuzumab with radiotherapy, reporting a median PFS of 5.8 months and median OS of 9.4 months (114). One- and two-year survival rates were 33.3 and 4.8%, respectively, with rare serious events including intratumoral bleeding. More recently, adding nimotuzumab to chemoradiation was feasible, with median OS 10.5 months and PFS 7.8 months, but did not meet a prespecified ORR-improvement threshold vs. historical data, supporting continued biomarker-guided optimization rather than routine adoption (118).

### Immunotherapies

Diffuse midline glioma is characterized as an immunologically ‘cold tumor’, with low lymphocytic infiltration and a microenvironment dominated by myeloid-lineage populations, which collectively constrains adaptive anti-tumor immunity and limits the efficacy of conventional immunotherapy approaches (119). These features highlight why DMG immunotherapy remains early-stage and why locoregional, antigen-directed strategies have become a central development focus (Table I).

### Chimeric antigen receptor (CAR)-T therapy

CAR-T-cell therapy aims to bypass limited endogenous T-cell priming by engineering T cells to recognize DMG-associated surface antigens, most prominently GD2 and B7-H3 (120). Upon antigen engagement, CAR signaling through CD3Z activates canonical T-cell effector programs, such as cytokine production and cytotoxicity, enabling direct killing of antigen-expressing tumor cells.

For GD2 targeting, first-in-human clinical experience demonstrated feasibility and early signals of activity, while highlighting neuroinflammation as an on-target risk that requires proactive monitoring and management (120). More recently, a phase I study of constitutively active IL-7 receptor-engineered GD2 CAR T cells (C7R-GD2.CART), further supported feasibility in H3K27-altered DMG, with tumor-inflammation-associated neurotoxicity and cytokine-release syndrome as key safety considerations and preliminary objective responses observed in a subset (95).

In parallel, B7-H3-directed CAR T cells have advanced through BrainChild-03 using repeated intra-cerebroventricular dosing; early DIPG evaluable cases showed no dose-limiting toxicities and evidence of local immune activation with a durable clinical/radiographic improvement in one patient (121). Updated Arm C results in DIPG expanded these observations by establishing feasibility of repetitive ICV dosing at higher planned dose regimens and providing benchmark survival and neurotoxicity profiles for future multi-site evaluation (122).

### Oncolytic virus DNX-2401

DNX-2401 is a conditionally replicating adenovirus designed to preferentially replicate in tumors with RB pathway dysregulation via a Δ24 deletion in E1A and its RGD modification can facilitate integrin-mediated entry (123).

In a phase I study of 12 newly diagnosed DMG/DIPG patients, a single stereotactic intra-tumoral infusion of DNX-2401 (1×10^10^ or 5×10^10^ viral particles) followed by radiotherapy achieved 92% disease control and a median OS of 17.8 months; 12- and 18-month OS were 75 and 50%, respectively (124). Correlative analysis supported treatment-associated immune activation, including increased T-cell-related activity, aligning with an oncolytic ‘viro-immunotherapy’ rationale in DMG.

### Cancer vaccine

Cancer vaccines aims to induce tumor antigen-specific adaptive immunity. In DMG, the shared clonal neo-antigen H3K27M has enabled peptide-vaccine strategies that predominantly prime mutation-specific CD4+ T-cell responses, with potential downstream support for cytotoxic effector programs and can increase immune infiltration in selected patients (125). Mechanistically, deeper immune profiling in a long-term recovered responder following H3K27M vaccination, identified broad HLA background coverage with both B- and T-cell responses, supporting the concept that vaccine-elicited anti-H3K27M immunity can be durable in selected cases (126,127).

The first-in-human H3K27M peptide vaccine (H3K27M-vac) study enrolled eight adults with H3K27M-altered DMG and showed favorable tolerability with mutation-specific immune responses dominated by CD4+ T cells; reported outcomes included median PFS 6.2 months and median OS 12.8 months (125). In younger patients, PNOC007 evaluated an HLA-restricted H3.3K27M peptide vaccine with adjuvant immunostimulation after radiotherapy and demonstrated immunogenicity with acceptable safety, reporting ~40% (DIPG) and ~39% (non-pontine DMG) 12-month OS benchmarks (128). INTERCEPT H3 is currently evaluating the use of H3K27M-vac concurrently with standard radiotherapy and in combination with the PD-L1 antibody atezolizumab for maintenance after radiotherapy to clarify its safety and immunogenicity (129).

## 7. Conclusion

Diffuse midline glioma, particularly the H3K27M-mutated subtype, remains a formidable CNS malignancy due to its unique molecular features and invasiveness. The present review emphasized the pivotal role of histone alterations, especially H3K27M, in reshaping chromatin states and gene expression programs that drive malignant proliferation, invasion and therapeutic resistance. Although surgery and radiotherapy remain foundational, their effect is constrained by anatomical inoperability and diffuse infiltration. Chemotherapy provides only a modest benefit and is frequently limited by resistance. By contrast, emerging targeted approaches, including ONC-201, panobinostat-based strategies and CDK4/6 inhibition combined with mTOR blockade, directly address molecular vulnerabilities, while early immunotherapy studies, including locoregional CAR-T platforms, DNX-2401 virotherapy and H3K27M-directed vaccination, illustrate feasibility and biological activity in selected settings.

Limitations of the current evidence base include small patient numbers, heterogeneous cohorts (age, tumor location, co-alterations and prior therapy) and variable drug-delivery strategies, which collectively limit cross-trial comparability and may inflate apparent signals in single-arm studies. Future progress will require biomarker-guided stratification, rational combination regimens aligned to DMG biology (including radiotherapy-integrated approaches) and delivery-optimized platforms that improve intra-tumoral exposure while maintaining neurological safety. Equally important, standardized response assessment and integrated correlative monitoring (molecular and immune profiling, longitudinal sampling, where feasible) should be embedded into multicenter trials to distinguish durable benefit from transient inflammatory effects and to enable definitive efficacy testing.

## Figures and Tables

**Table I.** Summary of clinical trials on chemotherapy, targeted therapy and immunotherapy in the treatment of DMG.

First author/s, year	Treatment type	Drug	Targets	Patients (n)	PFS (months)	mOS (months)	Other outcomes	(Refs.)
Bailey *et al*, 2013	Chemotherapy	Temozolomide	/	43	/	9.5	1-year OS ~35%; 2-year OS ~17%	(79)
Chassot *et al*, 2013		Temozolomide		22	7.5	11.7		(80)
Massimino *et al*, 2008		Cisplatin + Etoposide		62	7	/	1-year OS: 45%	(87)
Ono *et al*, 2021		Cisplatin/Etoposide		1	/	/	OS >6 years	(89)
Veldhuijzen van Zanten *et al*, 2017		Gemcitabine		9	4.8	8.7		(90)
Kilburn *et al*, 2018		Capecitabine		44	/	/	1-year PFS rate: 7.2%	(91)
Krystal *et al*, 2024		Irinotecan + Bevacizumab + Mebendazole		10	4.7	11.4		(93)
Lin *et al*, 2024		Cyclophosphamide + GD2.CART		11	/	/	7/11 neurological improvement	(95)
Venneti *et al*, 2023	Targeted Therapies	ONC-201	ClpP	30	3.4	9.3		(100)
Arrillaga-Romany *et al*, 2024		ONC-201	ClpP	477	/	/		(101)
Su *et al*, 2022		Vorinostat	HDAC	79	/	/	1-year PFS rate: 5.85%; 1-year OS rate:39.2%	(105)
Mueller *et al*, 2023		Panobinostat	HDAC	7	7	26.1		(104)
Neth *et al*, 2022		Panobinostat	HDAC	10	19	42		(106)
DeWire *et al*, 2020		Ribociclib	CDK4/6	10	/	16.1		(111)
DeWire *et al*, 2022		Ribociclib + Everolimus	CDK4/6 + mTOR	19	/	13.9		(112)
Fleischhack *et al*, 2019		Nimotuzumab	EGFR	42	5.8	9.4		(114)
Liu *et al*, 2025		Nimotuzumab	EGFR	48	7.8	10.5		(118)
Majzner *et al*, 2022	Immunotherapy	GD2-CAR T	/	4	/	/	3/4 Tumor volume reduction or stabilization	(120)
Lin *et al*, 2024		C7R-GD2.CART		11	/	/	Feasibility; on-target neuroinflammation/TIAN and CRS	(95)
Vitanza *et al*, 2025		B7-H3 CAR-T		21	/	/	Repetitive ICV dosing feasible; DLT reported (intratumoral hemorrhage)	(122)
Pérez-Larraya *et al*, 2022		DNX-2401		12	/	17.8		(124)
Grassl *et al*, 2023		H3K27M-vac		8	6.2	12.8		(125)
Mueller *et al*, 2020		H3.3K27M peptide vaccine		29 (A=19; B=10)	/	16.1 vs. 9.8		(128)

PFS, progression-free survival; mOS, median overall survival; ClpP, caseinolytic protease P; HDAC, histone deacetylase; mTOR, mechanistic target of rapamycin; EGFR, epidermal growth factor receptor; TIAN, tumor inflammation-associated neurotoxicity; CRS, cytokine release syndrome; ICV, intracerebroventricular; DLT, dose-limiting toxicity.

## Data Availability

Not applicable.
